# PBA Preferentially Impairs Cell Survival of Glioblastomas Carrying mutp53 by Reducing Its Expression Level, Stabilizing wtp53, Downregulating the Mevalonate Kinase and Dysregulating UPR

**DOI:** 10.3390/biom10040586

**Published:** 2020-04-10

**Authors:** Maria Anele Romeo, Maria Saveria Gilardini Montani, Rossella Benedetti, Alessia Garufi, Gabriella D’Orazi, Mara Cirone

**Affiliations:** 1 Department of Experimental Medicine, “Sapienza” University of Rome, Italy, Laboratory affiliated to Istituto Pasteur Italia-Fondazione Cenci Bolognetti, 00161 Rome, Italy; mariaanele.romeo@uniroma1.it (M.A.R.); mariasaveria.gilardinimontani@uniroma1.it (M.S.G.M.); benedetti.1589832@studenti.uniroma1.it (R.B.); 2 Department of Research, IRCCS Regina Elena National Cancer Institute, 00144 Rome, Italy; alessiagarufi@yahoo.it (A.G.); gdorazi@unich.it (G.D.); 3 Department of Medical, Oral and Biotechnological Sciences, University “G. d’Annunzio”, 66013 Chieti, Italy

**Keywords:** PBA, HDACi, glioblastoma, mutp53, mevalonate kinase, UPR

## Abstract

Phenylbutyrate (PBA) is a derivative of Butyric Acid (BA), which has the characteristics of being a histone deacetylase (HDAC) inhibitor and acting as a chemical chaperone. It has the potential to counteract a variety of different diseases, from neurodegeneration to cancer. In this study, we investigated the cytotoxic effect of PBA against glioblastoma cells carrying wt or mutant (mut) p53 and found that it exerted a higher cytotoxic effect against the latter in comparison with the former. This could be due to the downregulation of mutp53, to whose pro-survival effects cancer cells become addicted. In correlation with mutp53 reduction and wtp53 activation, PBA downregulated the expression level of mevalonate kinase (MVK), a key kinase of the mevalonate pathway strongly involved in cancer cell survival. Here we differentiated the chaperoning function of PBA from the others anti-cancer potentiality by comparing its effects to those exerted by NaB, another HDACi that derives from BA but, lacking the phenyl group, cannot act as a chemical chaperone. Interestingly, we observed that PBA induced a stronger cytotoxic effect compared to NaB against U373 cells as it skewed the Unfolded Protein Response (UPR) towards cell death induction, upregulating CHOP and downregulating BIP, and was more efficient in downregulating MVK. The findings of this study suggest that PBA represents a promising molecule against glioblastomas, especially those carrying mutp53, and its use, approved by FDA for urea cycle disorders, should be extended to the glioblastoma anticancer therapy.

## 1. Introduction

Phenylbutyrate (PBA) is an aromatic short-chain fatty acid known to exert multiple benefic effects, as it holds anti-inflammatory and anti-cancer properties. PBA and sodium butyrate (NaB) derive from modifications of Butyric Acid (BA) that, while maintaining the benefic pharmacologic properties of the molecule, increase its stability, thus rendering it more suitable for clinical use. Due to the addition of a phenyl group, PBA acquires also the capacity to act as chemical chaperone and may consequently help to restore the proper conformation of unfolded proteins, whose accumulation induces ER stress. ER stress/Unfolded Protein Response (UPR), usually activated in the cancer cells due to the intrinsic or extrinsic insults, may sustain cancer survival/chemoresistance [1]. Both PBA and NaB are histone deacetylase inhibitors (HDACis), and as such, they may hold a strong anti-cancer potential [2], also at sublethal doses. Indeed, together with genetic changes, post-translational modifications, including acetylation of histones and non-histone proteins, play a key role in cancerogenesis [3]. Interestingly PBA, being an intermediate metabolite of the phenylacetate, has been previously shown to reduce protein prenylation and cholesterol synthesis by inhibiting the mevalonate pathway [4]. Such an effect contributes to the PBA-mediated anti-cancer effect, particularly against gliomas [4] that relay more than other cancers on cholesterol metabolism [5]. Gliomas arise from oncogenic transformation of glial cells, more frequently astrocytes, and can behave either as low or as high aggressive cancers. The latter include the glioblastoma multiform (GBM), which represents the most common form of gliomas in the adult population. Its prognosis is worsened by the poor response to radio/chemotherapies, which renders even more urgent the search for new and more effective treatments able to interfere with its survival. P53, a protein that functions as a transcriptional regulator and plays a pivotal role in the controls of death/survival, is often deregulated in cancers and particularly in GBM. Indeed, as much as 94% of cell lines of GBM harbor p53 mutations which correlate with GBM aggressiveness [6]. The mutations occurring in the p53 encoding gene in GBM are mostly point mutations that affect the DNA binding domain of the protein. They may lead not only lose the oncosuppressor function of wtp53 but also lead to gain oncogenic functions (GOF), strongly contributing to GBM malignancy [6]. Indeed, mutp53 may cross-talk with several pro-oncogenic pathways such as the mevalonate and HSF/HSPs pathways to promote cancer cell survival [7]. Therapeutic approaches able to reduce the expression of mutp53 may represent a promising strategy for the treatment of GBM. Among the molecules regulating mutp53 stability, there are the HDACs, whose expression is frequently dysregulated in GBM. Importantly, the use of HDACis, besides reducing the acetylation of histones that leads to chromatin tightening and transcriptional repression, may also affect the acetylation and expression of non-histone proteins including mutp53 and the proteins involved in increasing its stability [8,9,10]. Interestingly PBA, in addition to being an HDACi, is a chemical chaperone that assists the folding of proteins and could facilitate the refolding of misfolded mutp53. The chaperoning strategy has been previously indicated as a possible strategy to rescue p53 mutant proteins [11]. Moreover, the chaperoning activity of PBA may influence the ER stress/UPR activation, orchestrated by IRE1alpha (inositol-requiring enzyme 1 alpha), PERK (PKR-like endoplasmic reticulum kinase) and ATF6 (Cyclic AMP-dependent transcription factor ATF-6 alpha), which regulate the balance between cell survival and cell death, mainly based on the expression of BIP (Binding Immunoglobulin Protein) and CHOP (C/EBP Homologous Protein), respectively. Although PBA has been reported to induce apoptosis in the glioma cell line LN-2299 by downregulating the anti-apoptotic bcl2 family proteins [12] the underlying mechanisms have not been investigated. In this study, the impact of PBA treatment on mutant and wtp53 expression, on the mevalonate pathway and ER stress/UPR was addressed as possible mechanisms of cell death induction in U373, T98 and U87, glioblastoma cell lines harboring mutant and wtp53, respectively.

## 2. Materials and Methods

### 2.1. Cell Cultures and Chemicals

U373, T98 and U87 (human glioblastoma cell lines with mutant and wild type p53) were grown in RPMI 1640 (Thermo Fisher Scientific), 10% Fetal Bovine Serum (FBS) (Corning), L-glutamine, streptomycin (100 μg/mL) (Corning) and penicillin (100 U/mL) (Corning) in 5% CO2 at 37 °C. Cells were always detached using Trypsin-EDTA solution (Biological Industries, Cromwell, CT, USA). 4-phenylbutyrate (PBA) and sodium butyrate (NaB) were purchased from Sigma-Aldrich.

### 2.2. Trypan Blue Exclusion Assay

U373, T98 and U87 cells were plated in 6-well plates at a density of 2×10^5^ cells/well. The following day, when the cells were in the exponential growth phase, cells were treated with PBA at 7.5 and 15 mM or NaB at 7.5 and 15 mM. After 24 h of culture, a trypan blue (Sigma-Aldrich) exclusion assay was performed to test cell viability. Cells were counted by light microscopy using a Neubauer emocytometer. The experiments were performed in triplicate and repeated at least three times.

### 2.3. MTT Assay

U373, T98 and U87 cells plated in a 96-well plate at 1×10^4^ cells/well were treated with PBA (7.5 mM, 15 mM) or left untreated. The following day, 20 μL of 5 mg/mL MTT (Sigma Diagnostic, St. Louis, MO, USA) in PBS 1× was added to each well for 4 h at 37 °C. The formazan crystals were dissolved in 100 μL anhydrous isopropanol with 0.1 N HCl (Sigma Diagnostic, St. Louis, MO, USA). The optical density was determined with a microculture plate reader (BIO-RAD MICROPLATE READER) at 590 nm. Each assay was performed in triplicate. Absorbance values were normalized to the values for the untreated cells to determine the percentage of survival. The experiments were performed in triplicate and repeated at least three times.

### 2.4. p53 Silencing

1.5 × 10^6^ U373 cells were transfected with empty vector or sip53 plasmid for p53 knockdown by electroporation using the Bio-Rad Pulse Controller at 180 Volts, according to the manufacturer’s instructions, and cultured in 24-well plates. After 24 h, cells were treated or not with PBA (7.5 mM) for an additional 24 h. After that period, a trypan blue assay was performed, cells were lysated and protein extracts were subjected to Western blot analysis.

### 2.5. Western Blot Analysis

1×10^6^ cells were washed with PBS and lysed in a RIPA buffer containing 150 mM NaCl, 1% NP-40 (Calbiochem), 50 mM Tris-HCl, pH 8, 0.5% deoxcycholic acid (SIGMA), 0.1% SDS, protease and phosphatase inhibitors. Twelve grams of protein lysates were subjected to protein electrophoresis on 4%–12% NuPage Bis-Tris gels (Sigma Aldrich), as previously described (Ref Capsaicin Garufi). The gels were blotted on nitrocellulose membrane (Biorad) for 1 hr in Tris-Glycine buffer. The membranes were blocked in PBS 0.1% Tween20 solution containing 3% of BSA, probed with specific antibodies and developed using ECL Blotting Substrate (Advansta).

### 2.6. Densitometric Analysis

The quantification of protein bands was performed by densitometric analysis using the Image J software (1.47 version, NIH, Bethesda, MD, USA), which was downloaded from NIH website (http://imagej.nih.gov).

### 2.7. Antibodies

To evaluate the expression of proteins, we used the following antibodies: mouse monoclonal anti-PARP1 (1:100) (Santa Cruz Biotechnology Inc.), mouse monoclonal anti-p53 (1:100) (clone DO-1, Santa Cruz Biotechnology Inc.), mouse monoclonal anti-p21 (1:100) (Santa Cruz Biotechnology Inc.), mouse monoclonal anti-MVK (1:100) (Santa Cruz Biotechnology Inc.), mouse monoclonal anti-pERK (1:200) (Santa Cruz Biotechnology Inc.), rabbit polyclonal anti-ERK1 and anti-ERK2 (1:200) (Santa Cruz Biotechnology Inc.), rabbit polyclonal anti-BIP (1:1000) (Cell Signaling) and mouse monoclonal anti-CHOP (1:100) (Santa Cruz Biotechnology Inc.). Mouse monoclonal anti-β-actin (1:10,000) (Novus Biological) was used as loading control. The goat anti-mouse IgG-Horseradish Peroxidase (Santa Cruz Biotechnology Inc.) and goat anti-rabbit IgG-HRP (Santa Cruz Biotechnology Inc.) were used as secondary antibodies. All the primary and secondary antibodies were diluted in PBS—0.1% Tween20 solution containing 3% of BSA (SERVA).

### 2.8. Statistical Analysis

Results are represented by the mean ± standard deviation (SD) of at least three independent experiments, and a two-tailed Student’s *t*-test was used to demonstrate statistical significance. Difference was considered as statistically significant when *p*-value was at least <0.05.

## 3. Results

### 3.1. PBA Reduces Cell Survival of U373 and T98 Glioblastoma Cells Harboring mutp53 More Efficiently than U87 wtp53-Carrying Glioblastoma Cells

**Figure 1:**
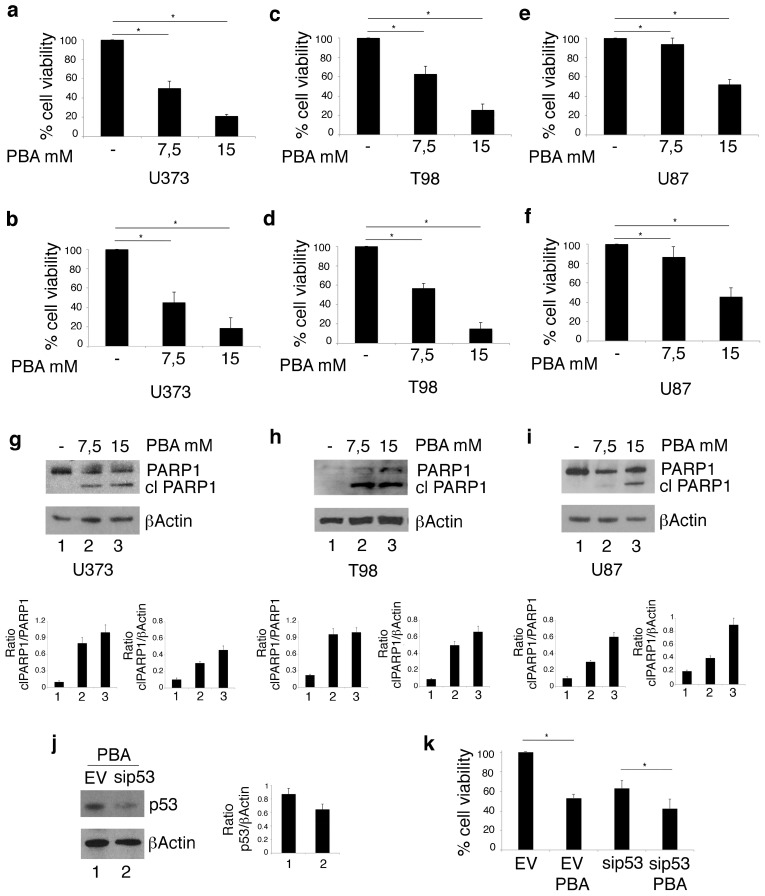
Phenylbutyrate (PBA) reduces cell survival and induces cell death in U373 and T98 glioblastoma cells more efficiently than in U87 glioblastoma cells. After 24 h of culture in the absence or in the presence of 7.5 mM or 15 mM PBA, cell survival of U373, T98 and U87 cell lines was evaluated by trypan blue exclusion assay (**a**, **c** and **e** respectively) and by MTT assay (**b**, **d** and **f** respectively). The histograms represent the mean plus SD of more than 3 experiments * *p*-value < 0.05; PARP1 cleavage (clPARP1) was also evaluated in these cells (**g**, **h** and **i** respectively); PBA cytotoxicity was also evaluated by trypan blue exclusion assay in U373, in which p53 was silenced (**j**, **k** and **l**). βActin was used as loading control. One representative experiment out of 3 is shown. The histograms (**j**) represent the mean plus SD of the densitometric analysis of the ratio of clPARP1/βActin of 3 different experiments. In l, histograms represent the inhibition of viability of PBA-treated EV and sip53 U373 cells expressed as % on each control. *p-value < 0.05.

U373, T98 cells harboring mutp53 and U87 carrying wtp53 were treated with PBA for 24 h. As evaluated by trypan blue (Figure 1a,c,e) and MTT (Figure 1b,d,f) assays, cell survival was reduced in a dose-dependent fashion in all cell lines, although U87 cells were less susceptible to such treatment in comparison to U373 and T98. These findings were confirmed by Western blot analysis showing that the cleavage of PARP1, observed at 7.5 mM dose of PBA in U373 and T98 cells (Figure 1e), was detectable only at 15 mM in U87 cells (Figure 1f). The latter experiment also indicates that PBA triggered an apoptotic cell death in both glioblastoma cell lines, although more efficiently in the mutp53-carrying U373 and T98 cells. To investigate whether mutp53 render cancer cells more susceptible to PBA treatment, we silenced p53 (Figure 1j) and found that PBA cytotoxicity was reduced in p53-silenced cells in comparison to the empty-vector treated control cells (Figure 1k,l).

### 3.2. PBA Reduces mutp53 Expression in U373 Cells and Activates wtp53 in U373 and U87 Cells, Reducing the MVK of Mevalonate Pathway

**Figure 2:**
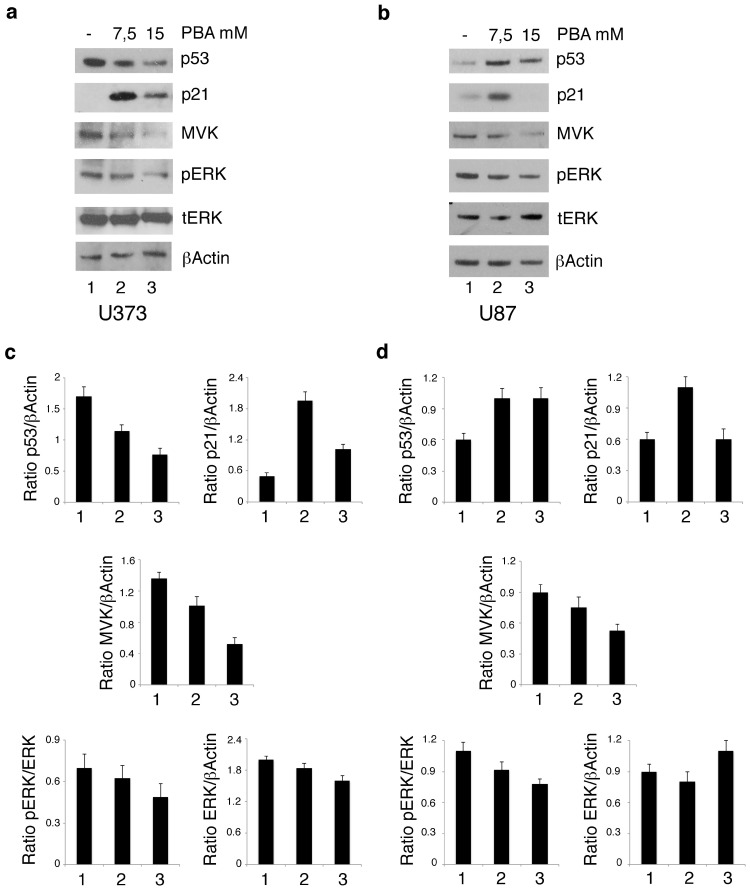
PBA reduces mutp53 expression in U373 cells and activates wt p53 in U373 and U87 cells, reducing the MVK of mevalonate pathway. U373 (**a**) and U87 (**b**) cell lines, cultured for 24 h in the absence and in the presence of 7.5 mM or 15 mM PBA, were analyzed by Western blot for p53, p21, MVK, pERK and ERK protein expression. βActin was used as loading control. One representative experiment out of 3 is shown. In **c** and **d**, the histograms represent the mean plus SD of the densitometric analysis of the ratio of p53, p21, MVK, pERK and ERK on the loading control of 3 different experiments.

As HDACis have been previously reported to reduce mutp53 in several cancer cell types [13,14] and the reduction of mutant p53 represents a therapeutic strategy against gliomas [6], we next evaluated the mutp53 expression level in U373 cells undergoing PBA treatment. As shown in Figure 2a, mutp53 was reduced by PBA in a dose-dependent fashion. On the other hand, in U87 cells that harbor wtp53, PBA treatment increased its expression (Figure 2b) and this effect could correlate with induction cell death also in these cells, although to a lesser extent compared to U373. Interestingly, we found that one of the targets of wtp53, namely p21, was upregulated by PBA in both cell lines (Figure 2a,b), suggesting, in the case of U373 cells, that PBA, besides reducing mutp53, was able to stabilize mut53 towards a wt conformation. Previous studies have indicated that acetylation status may influence the activity of wtp53 [15], and this could underlie the effect induced by PBA on this protein. Mutp53 and wtp53 have been reported to control in an opposite fashion the transcription of enzymes of the mevalonate pathway [16,17] involved in the survival of cancer cells and particularly of glioma cells [4]. Therefore, we next investigated the expression of mevalonate kinase (MVK) in both U373 and U87 cell lines. MVK is the first enzyme to follow 3-hydroxy-3-methyl-glutaryl-CoA reductase (HMG-CoA reductase) in the mevalonate pathway, and its deficiency leads to autoinflammatory disorders [18]. As shown in Figure 2a,b, MVK expression was reduced in both cell lines following PBA treatment, an effect occurring in a dose-dependent fashion in U373 cells but observed only at the highest dose in U87 cells, according to the dose required to reduce their cell survival. As the mevalonate pathway sustains oncogenic pathways such as ERK1/2 (Extracellular signal-regulated kinases) by promoting the prenylation of Ras protein [19,20], we next assessed the phosphorylation status of ERK 1/2 and found that its phosphorylation was reduced in correlation with MVK downregulation following PBA treatment (Figure 2a,b). Altogether, these results suggest that PBA downregulates mutp53 and activates wtp53 both in wt and mutp53-carrying cells and that such effects correlated with the reduced expression of MVK of the mevalonate pathway and ERK1/2-reduced phosphorylation in glioblastoma cells.

### 3.3. The Chaperoning Function of PBA Does Not Affect mutp53 Downregulation and Its Stabilization Toward a Wild-Type Conformation in U373 Cells

**Figure 3:**
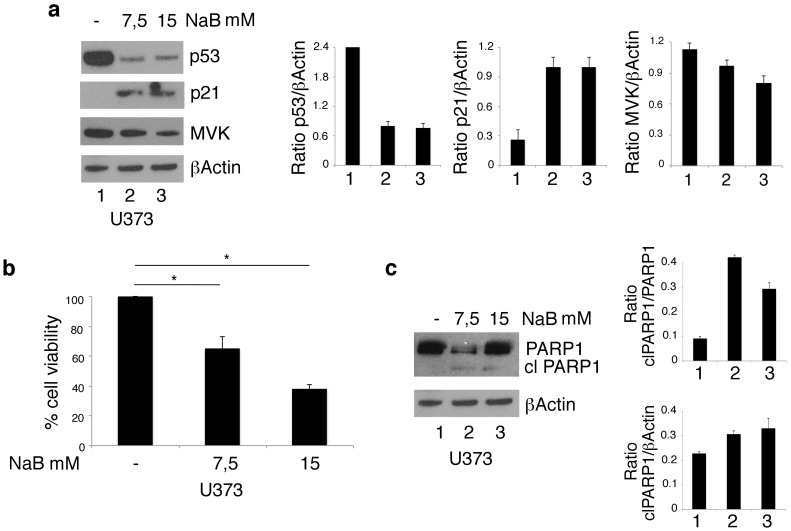
NaB downregulates mutp53 expression level and reduces cell viability in U373 glioblastoma cells. p53 and p21 protein expression (**a**) was analyzed by Western blot of U373 cell line cultured in the absence or in the presence of 7.5 or 15 mM NaB for 24 h. βActin was used as loading control. One representative experiment out of 3 is shown. The histograms represent the mean plus SD of the densitometric analysis of the ratio p53/βActin[SL1] and p21/βActin; cell survival of U373 cell line cultured in the absence or in the presence of 7.5 or 15 mM NaB for 24 h was evaluated by trypan blue exclusion assay (**b**). The histograms represent the mean plus SD of more than 3 experiments * *p*-value < 0.05; (**c**) cleavage of PARP1 (clPARP1) was analyzed by Western blot of U373 cell line cultured in the absence or in the presence of 7.5 or 15 mM NaB for 24 h. βActin was used as loading control. One representative experiment out of 3 is shown. The histograms represent the mean plus SD of the densitometric analysis of the ratio of clPARP1/PARP1and clPARP1/βActin.

The stabilization of mutp53 toward a wild-type conformation in glioblastoma cells treated by PBA could contribute to its chaperoning function. To understand whether this could be the case, we evaluated the expression level of mutp53 and the expression of the wtp53 target p21 in U373 cells treated with NaB, another HDACi that also derives from BA and shares with PBA the property of being an HDACi but not the capacity to act as a chemical chaperone. We found that NaB very efficiently reduced mutp53 expression level and stabilized it toward a wild type conformation, as demonstrated by the upregulation of p21 (Figure 3a). These results indicate that PBA’s chaperoning function was not involved in these effects, which were rather due to its HDAC inhibitory activity. However, to evaluate whether the chaperoning function of PBA could play a role in its mediated cytotoxic effect, we investigated the cytotoxicity of NaB against U373 cells. We found that NaB, used at the same doses (Figure 1a,b), was less cytotoxic than PBA, as indicated by trypan blue assay (Figure 3b) and the analysis of PARP1 cleavage (Figure 3c).

### 3.4. PBA and NaB Differently Affect the Expression of the Pro-Survival UPR Molecules in U373 Cells

**Figure 4:**
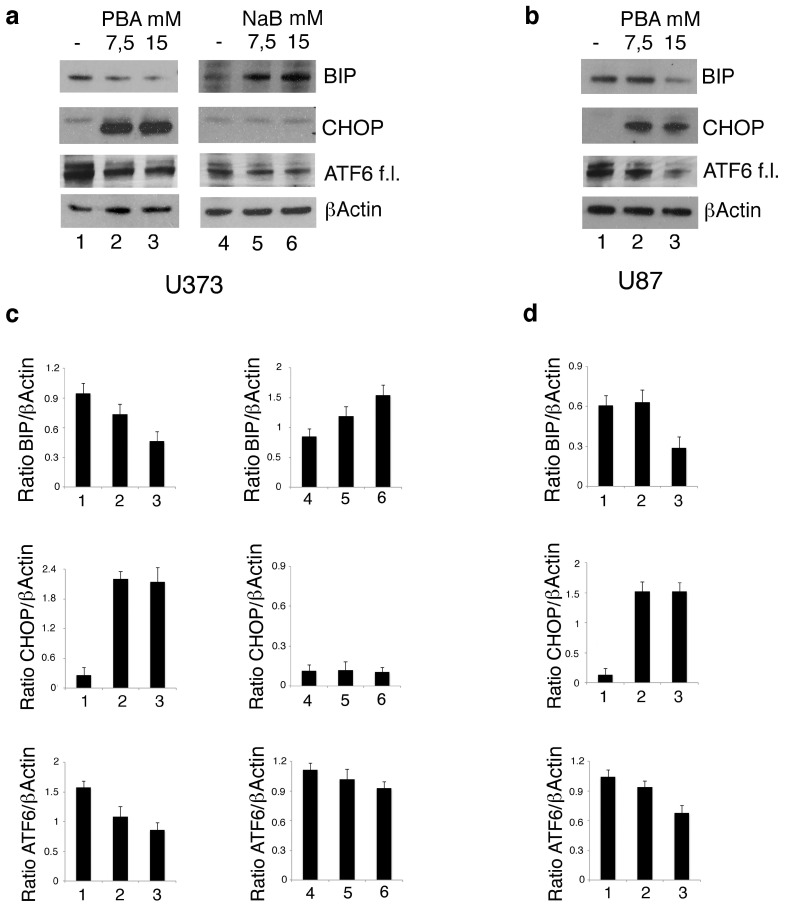
PBA and NaB differently affect the expression of the Unfolded Protein Response (UPR) molecules in U373 cells. U373 (**a**) cell line, cultured in the absence and in the presence of 7.5 mM or 15 mM PBA or in the presence of 7.5 mM or 15 mM NaB, and U87 (**b**) cultured in the absence and in the presence of 7.5 mM or 15 mM PBA for 24 h, were analyzed by Western blot for ATF6, BIP and CHOP expression. βActin was used as loading control. One representative experiment out of 3 is shown. The histograms represent the mean plus SD of the densitometric analysis of the ratio of ATF6, BIP and CHOP on βActin of 3 different experiments performed with U373 (**c**) and U87 (**d**).

As PBA is a chemical chaperone, we then asked whether its higher cytotoxic effect compares to NaB could be due to the reduction of ER stress and to an altered expression of the pro-survival and pro-death molecules of UPR. To this aim, we evaluated the expression level of ATF6, BIP and CHOP, UPR molecules. As shown in Figure 4a, PBA activated ATF6, as indicated by the reduced expression of its full-length form, reduced BIP and strongly upregulated CHOP in U373 cells. In the same cells, NaB treatment activated ATF6 to a lesser extent, did not affect CHOP expression and upregulated BIP. The different regulation of UPR in terms of BIP and CHOP expression by PBA and NaB in U373 could underlie the different cytotoxicity of these drugs against these cells. Finally, as mutp53 may itself strongly modulate the stress response in cancer cells [7,21], we evaluated ATF6, BIP and CHOP expression level also in U87 cells carrying wtp53 following PBA treatment. As shown in Figure 4b, PBA treatment activated ATF6 and downregulated BIP only at the highest dose, the same ability to induce the cytotoxic effect in these cells (Figure 1b), while CHOP was upregulated at both doses. These results suggest that, in cells harboring wtp53, the effect of PBA on UPR was less evident than in mutp53 carrying cancer cells, and this effect correlated with the lower cytotoxic effect observed in U87 cells.

## 4. Discussion

Acetylation is one of the epigenetic modifications that support survival and progression of cancers. However, genetic changes such as those affecting the p53 oncosuppressor gene are also very common in cancers and, especially in the case of gliomas, correlate with their malignancy and aggressiveness [6]. In this study, we show that PBA, an HDACi inhibiting Class I and II HDACs exerted a strong cytotoxic effect against U373 and T98 glioblastoma cells harboring mutp53, and such cytotoxicity correlated with the downregulation of mtp53 expression level. Interestingly, the use of HDACis has been previously reported to affect mutp53 stability [14]. PBA was also able to activate wtp53 in U373 cells, an effect that was also induced in wt-p53-carrying U87 cells, although this cell line was more resistant to the PBA treatment. This is maybe due to the fact that U373, like other cancer cells harboring oncogene or oncosuppressor gene mutations, are addicted to the pro-survival effects of mutp53, and this may turn out to be an Achille’s heel to be exploited in anticancer therapy [21]. Similarly to PBA, it has been previously described that another HDACi, the suberoylanilide hydroxamic acid (SAHA), preferentially killed mutp53-carrying cancer cells [14]. In the case of SAHA, the reduction of mutp53 the expression level was due to the inhibition of HDAC6 that leads to the hyperacetylation of HSP90, preventing its capacity to stabilize mutp53. PBA seems to be not able to inhibit HDAC6, and thus it may influence mut53 and wtp53 expression level by directly affecting the acetylation status of p53 or that of other p53 stabilizing molecules. However, differently from SAHA, PBA, being a chemical chaperone, could help the proper folding of mut53, even if this property seems not to be involved in the reduction of mutp53 and to its stabilization toward a wild type conformation in U373 cells. Indeed, these effects were also mediated by NaB, an HDACi that also derives from BA but lacks the phenyl group and thus the chaperoning function. Interestingly, in this study, we found that PBA, in correlation with mutp53 reduction or wtp53 reactivation, reduced MVK, a kinase of the mevalonate pathway that sustains cancer cells’ survival. Accordingly, previous studies have reported that mutp53 [16] and wt p53 [17] may oppositely affect SREBPs, the master regulators of the transcription of enzymes of the mevalonate pathway. Furthermore, a positive feedback loop has been reported to occur between mutp53 and the mevalonate pathway as mutp53 activates the transcription of the mevalonate pathway enzymes and the latter in turn promotes the stability of mutp53 by sustaining its interaction with DNAJA1 Hsp40 [22] or with HDAC6/Hsp90 [7,10]. The capacity to stabilize mutp53 has been confirmed in this study by the use of lovastatin that reduced mutp53 expression level in U373 cells (data not shown). The targeting of the mevalonate pathway is emerging as a promising strategy to counteract several diseases including cancer [18,23]. Of note, mevalonate is not the only pathway with which mutp53 establishes a criminal alliance to promote cancer cell survival/progression/chemoresistance [7,24]; therefore its downregulation by PBA may interfere with a variety of other oncogenic pathways. Also important is that ERK1/2 phosphorylation was reduced by PBA, as this MAPK is at a center of a network that controls cell proliferation, apoptosis, differentiation and stress response in glioblastoma cells. In this study, we also observed that PBA reduced the expression of the pro-survival UPR molecule BIP and upregulated the pro-mortem UPR molecule CHOP in U373 more efficiently than NaB and that the latter was also less efficient in downregulating MVK, in correlation with its lower cytotoxic effect. Regarding the impact of PBA on UPR in U87 carrying wtp53, the finding that the downregulation of BIP occurred only at a higher dose in these cells could be explained by the absence of mutp53 that has been reported to help cancer cells to survive and adapt to conditions of chronic stress, due to intrinsic and extrinsic insults [7,21,25].

## 5. Conclusions

In conclusion, this study suggests that PBA represents a promising therapeutic strategy against malignant glioblastomas, especially those that harbor mutp53 and have a more aggressive behavior than those carrying wtp53. The use of this drug, already approved by FDA for the treatment of urea cycle disorders, should be extended also to the treatment of glioblastomas, also considering that it is able to penetrate the brain–blood barrier, which helps to ameliorate the neurodegeneration and that it has not reported to exert important side effects or even ameliorate several physiological functions [26].
